# A continuous myofibroblast precursor cell line from the tail muscle of Australasian snapper (*Chrysophrys auratus*) that responds to transforming growth factor beta and fibroblast growth factor

**DOI:** 10.1007/s11626-022-00734-2

**Published:** 2022-11-15

**Authors:** Gavril L. W. Chong, Björn Böhmert, Lucy E. J. Lee, Niels C. Bols, Georgina C. Dowd

**Affiliations:** 1 The New Zealand Institute for Plant and Food Research Ltd, Nelson Research Centre, 293 Akersten Street, Nelson, 7010 New Zealand; 2 Faculty of Science, University of the Fraser Valley, Abbotsford, BC V2S 7M8 Canada; 3 Department of Biology, University of Waterloo, Waterloo, ON N2L 3G1 Canada

**Keywords:** Australasian snapper, *Chrysophrys auratus*, *Pagrus auratus*, Cell line, Growth factors, Myofibroblast, Actin bundling, Collagen, Extracellular matrix, Mesenchymal stem cells

## Abstract

*Chrysophrys auratus* (Australasian snapper) is one of the largest and most valuable finfish from capture fisheries in New Zealand, yet no cell lines from this species are reported in the scientific literature. Here, we describe a muscle-derived cell line initiated from the tail of a juvenile snapper which has been designated CAtmus1PFR (*Chrysophrys auratus,* tail muscle, Plant & Food Research)*.* The cell line has been passaged over 100 times in 3 years and is considered immortal. Cells are reliant on serum supplementation for proliferation and exhibit a broad thermal profile comparable to the eurythermic nature of *C. auratus* in vivo. The impact of exogenous growth factors, including insulin-like growth factors I and II (IGF-I and IGF-II), basic fibroblast growth factor (bFGF), and transforming growth factor beta (TGFβ), on cell morphology and proliferation was investigated. Insulin-like growth factors acted as mitogens and had minimal effect on cell morphology. TGFβ exposure resulted in CAtmus1PFR exhibiting a myofibroblast morphology becoming enlarged with actin bundling. This differentiation was confirmed through the expression of smooth muscle actin (*sma*), an increase in type 1 collagen (*col1a*) expression, and a loss of motility. Expression of *col1a* and *sma* was decreased when cells were exposed to bFGF, and no actin bundling was observed. These data indicate that CAtmus1PFR may be myofibroblastic precursor cells descending from mesenchymal progenitor cells present in the tail muscle myosepta.

**Supplementary Information:**

The online version contains supplementary material available at 10.1007/s11626-022-00734-2.

## Introduction

The Australasian snapper (*Chrysophrys auratus*, formerly known as *Pagrus auratus*) is a marine teleost which is found in the coastal waters of Australia and New Zealand (Parsons *et al.* 2014). The species has economic significance in New Zealand, representing one of the largest capture fisheries in the country. In addition, snapper, or tāmure, have cultural significance to the indigenous people of New Zealand (Māori) as well as being a popular recreational fishery (Parsons *et al.* 2014). These factors have made snapper a prime candidate for aquaculture. A selective breeding programme to produce snapper strains that are adapted to domestication is ongoing in New Zealand (Ashton *et al.* 2019; Wellenreuther *et al.* 2019). Despite the significance of this species, there are no cell lines derived from *C. auratus* tissue, including muscle. As fish strains that exhibit more rapid muscle growth are desirable for commercial purposes (Olesen *et al.* 2003; Sandoval-Castillo *et al.* 2022), availability of muscle-derived cell lines could assist aquaculture of snapper through fundamental understanding of muscle growth, development, and repair in vitro, but would also provide a platform for controlled analysis of single or multiple parameters at a time, e.g. temperature, salinity, and nutrients. These types of analyses can be challenging when dealing with live organisms in complex aquatic environments. A well-characterised cell line from one species has the potential to act as a model system for other related species. There are also possible applications of cell lines beyond traditional aquaculture in the field of cellular agriculture (Rubio *et al.* 2019).

Fish skeletal muscle is composed mainly of muscle fibres which form through recruitment of new fibres (hyperplasia) or growth of existing fibres (hypertrophy) (Zimmerman and Lowery 1999; Ruparelia *et al.* 2020). The dimensions and distribution of fish muscle fibres, coupled with the extent of intramuscular fat, and the composition of intramuscular connective tissue are important factors which affect the final texture and quality of the fish flesh (Kiessling *et al.* 2006; Listrat *et al.* 2016). Despite this, description of specific cell types beyond fibre-forming stem/satellite cells isolated from fish embryos or skeletal muscle (Gignac *et al.* 2014; Peng *et al.* 2016; Sultan *et al.* 2021) is limited, particularly in comparison to mammalian systems where vasculature-associated stem cells including mesoangioblasts and pericytes have been described, in addition to interstitial cells such as fibro-adipogenic progenitor (FAP) cells and PW1-positive interstitial cells (PICs) (Cottle *et al.* 2017; Nassari *et al.* 2017; Rubenstein *et al.* 2020). The role of these and other progenitor cells of the myoseptum is essentially unexplored in fish muscle (Charvet *et al.* 2011; Bricard *et al.* 2014; Ruparelia *et al.* 2020).

The fish skeletal muscle invitrome (i.e. a grouping of cell lines around a theme or category (Bols *et al.* 2017)) is extremely limited. Currently, approximately 26 skeletal muscle cell lines from 20 fish species have been described in the scientific literature (Cellosaurus 2022). This number of species represents less than 0.04% of all known fish species (> 34,000) (www.fishbase.org). Of these cell lines, the majority are classified by cell morphology, mainly as fibroblastic or epithelial, with few progressing characterisation to describe a specific cell type (Fernandez *et al.* 1993; Zhao *et al.* 2003; Zhao and Lu 2006; Rougee *et al.* 2007; Lai *et al.* 2008). A key challenge in establishing well-characterised muscle (or any other tissue)-derived fish cell lines is the lack of resources such as validated, species-specific antibodies, detailed knowledge of cell lineages that exist in fish muscle, a lack of fundamental knowledge on the application of cell sorting technologies specifically for fish cells, and the huge breadth of species making transfer of technology and knowledge challenging (Rubio *et al.* 2019; Potter *et al.* 2020). In the absence of these resources, one way of determining a specific cell type is to investigate the genetic, physical, and biochemical responses of cells to various growth factors or cytokines, as their impacts on skeletal muscle development, homeostasis, and repair are reasonably well understood across species (Syverud *et al.* 2016, Vélez *et al.* 2017; Duran *et al.* 2022) . Fibroblast growth factor (FGF), transforming growth factor β (TGFβ), and insulin-like growth factors (IGFs) are some of the most influential cytokines involved in these processes (Dayton and Hathaway 1991; Syverud *et al.* 2016).

FGFs have been shown to promote proliferation of activated myogenic stem cells, suppressing their terminal differentiation (Dayton and Hathaway 1991; Fox and Swain 1993). In addition, FGFs have been shown to enhance the proliferation of fibroblasts and promote the formation of new blood vessels (Floss *et al.* 1997; Kastner *et al.* 2000). Myogenic stem cell differentiation is inhibited by TGFβ through the inhibition of myogenic regulators MyoD and myogenin (Liu *et al.* 2001). TGFβ regulates the phenotype and function of fibroblasts and plays a role in myofibroblast transdifferentiation (Krummel *et al.* 1988). This process promotes expression of extracellular matrix and profibrotic genes such as type 1 collagen (*col1a*) (Ignotz and Massagué 1986; Grande *et al.* 1997) and connective tissue growth factor (*ctgf*) (Leask *et al.* 2009; Tsai *et al.* 2018). In some situations, excessive TGFβ-induced deposition of extracellular matrix components can lead to fibrosis, a condition which severely impairs muscle function via reduced mobility and contractile function (Mann *et al.* 2011). Fibrosis in fish heart, liver, and muscle has been reported in vivo (Arana *et al.* 2014; Keen *et al.* 2015; Lin *et al.* 2022). IGFs stimulate nutrient uptake, enhance myoblast proliferation, and increase protein synthesis across a number of species (Negatu and Meier 1995; Rius-Francino *et al.* 2011; Castillo *et al.* 2004; Codina *et al.* 2008; Díaz *et al.* 2009; Gabillard *et al.* 2010; Montserrat *et al.* 2012). There is also evidence that exogenous addition of IGF-I to primary myocytes derived from gilthead seabream, a closely related species to the Australasian snapper, resulted in increased expression of myogenin, a transcriptional factor involved in myogenesis, indicating that IGF may also play a role in the differentiation process (Vélez *et al.* 2014).

Here we report on the first *Chrysophrys auratus–*derived cell line, among several others currently in development at Plant & Food Research (Nelson, New Zealand). This cell line will be identified as *CAtmus1PFR* (***C****hrysophrys* ***a****uratus* **t**ail **mus**cle **1** **P**lant & **F**ood **R**esearch—species; tissue; institute affiliation). Our findings indicate that this cell line is a myofibroblastic progenitor cell derived from the non-myogenic line of mesodermal progenitor cells.

## Materials and methods

### Cell isolation and primary culture

**Figure 1.**
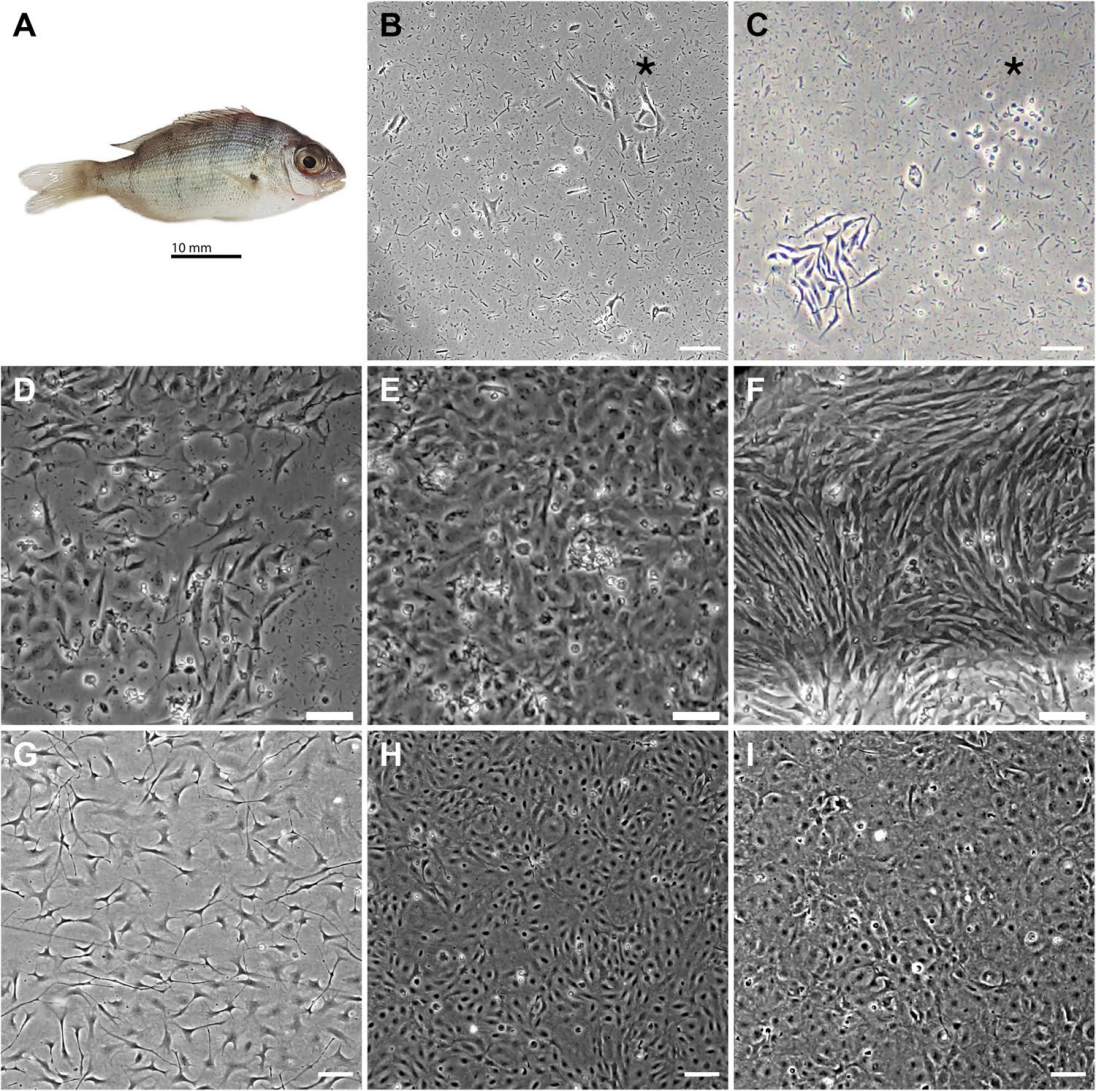
Establishment of CAtmus1PFR muscle-derived cell line from *Chrysophrys auratus.* (*A*) Snapper from where CAtmus1PFR was derived. (*B*) Cells 9 d after isolation. (*C*) Early cell death. (*D*) Additional cell types (*E–F*) Cells with various morphologies after 1 month in culture. (*G*) Fibrous morphology of CAtmus1PFR when sub confluent. (*H*) Confluent morphology and (*I*) Confluent cells with sub-population of large rounded cells. * indicates same position on micrographs (*B*) and (*C*). All *scale bars* are 100 µm.

A single healthy juvenile snapper (*Chrysophrys auratus*) bred at The New Zealand Institute for Plant and Food Research Limited was euthanised with an overdose of anaesthetic (60 ppm AQUI-S, Lower Hut, New Zealand). The fish weighed 1.45 g and was 4.5 cm in length from nose to peduncle (Fig. 1*A*). The specimen was kept on ice for 30 min before processing for cell culture. The fish was surface sterilised with 70% ethanol and approximately 1 cm^3^ of de-skinned muscle tissue was excised from the tail peduncle. The muscle segment was washed extensively in Hanks’ balanced salt solution (HBSS, Sigma Aldrich, St Louis, MO) supplemented with 100 units mL^−1^ penicillin and 100 µg mL^−1^ streptomycin (1 × P/S) (Sigma-Aldrich). Using sterile scalpel blades, the tissue was minced into small fragments which were further dissociated enzymatically by incubation with 1 mg mL^−1^ collagenase in HBSS at 18 °C for 4 h. The dissociated tissue fragment was centrifuged at 95 × *g* for 5 min at room temperature (Hettich 320R), after which the supernatant was removed and the remaining cell pellet resuspended in 2 mL Leibovitz’s L-15 (L-15, Gibco, Grand Island, NY) medium supplemented with 25% FBS (Gibco) and 1 × P/S. The resuspended cells were added to a 25-cm^2^ flask (Falcon, Corning, NY) which was incubated in the dark, in ambient air and temperature (approximately 18 °C). Twenty-four hours later, media containing unattached cells and debris was removed and fresh media added (L-15, 25% FBS, 1 × P/S). After about 6 weeks in culture, the original T25 flask reached confluency, and was passaged to a fresh flask as indicated below, and the serum concentration was reduced to 20%. Once the cells had been passaged four times, the serum concentration was reduced to 10%. Cells were monitored routinely using an inverted microscope (CKX53, Olympus, Tokyo, Japan).

### Cell line maintenance

Cells were routinely cultured in standard non-vented tissue culture flasks (Nunc EasYFlask, Thermo Fisher Scientific, Waltham, MA) at 18 °C, in the dark, in ambient air. L-15 medium supplemented with 10% FBS and 1 × P/S was used for routine culture. On reaching confluency, cells were detached from culture vessels by exposure to 1 × TrypLE Express (Gibco) for 5–6 min followed by centrifugation at 95 × *g* for 5 min. Spent medium was removed, and the cells were reseeded in fresh media at 0.133 mL media cm^2-^ (i.e. 10 mL for a 75-cm^2^ flask). For cryopreservation, cells were pelleted (95 × *g* for 5 min) and resuspended in 0.5 mL L-15 media (10% FBS). An equal volume of freezing media (L-15 with 20% dimethyl sulfoxide (DMSO)) was added for a final cell concentration of 1 × 10^6^ cells mL^−1^ and a final DMSO concentration of 10%. Cells were stored at − 80 °C for a minimum of 48 h before long-term storage in the vapour phase of liquid nitrogen.

### Mycoplasma detection

Cells at passage 20, 28, and 48 h were cultured to confluency in the absence of antibiotics for 2 weeks. The presence of contaminating mycoplasma species was monitored using the Lookout® Mycoplasma PCR Detection Kit according to the manufacturer’s instructions (Sigma-Aldrich).

### DNA barcoding

Cell culture provenance was verified by DNA barcoding using universal non-specific primers targeting conserved sequences of cytochrome c oxidase subunit I (*COI*) and 16 s rRNA, and comparison to amplicons of the same genes of gDNA extracted from whole fish. DNA was isolated from CAtmus1PFR using a Purelink™ Genomic DNA Extraction Kit (Invitrogen, Carlsbad, CA) according to the manufacturer’s recommendations. gDNA from a fin clip of *C. auratus* was extracted using the method described previously by Ashton *et al.* (2019). Briefly, a fresh fin clip was placed into chilled 96% ethanol followed by heating to 80 °C for 5 min. The sample was stored at − 20 °C until additional processing occurred. Approximately 20 mg of the fin clip was digested by incubation in extraction buffer (0.4 M NaCl, 10 mM Tris–HCl pH 8.0, 2 mM EDTA pH 8.0) with 2% SDS (final concentration) at 80 °C for 5 min followed by rapid cooling on ice. The sample was digested with proteinase K (400 µg mL^−1^ final concentration) for 1.5 h at 56 °C, after which the sample was centrifuged at 13,680 × *g* for 15 min. Following treatment with 5 M NaCl and centrifugation, RNAse A (100 µg mL^−1^) was added to remove contaminating RNA. DNA was precipitated with isopropanol overnight at − 20 °C. Subsequent ethanol washes followed by air-drying yielded purified gDNA.

*16 s rRNA* and *COI* amplifications from gDNA extracted from the fin clip and cell line were performed using BIOTAQ™ DNA Polymerase (Meridian Bioscience, Cincinnati, OH) according to the manufacturer’s instructions. Forward and reverse primers (Table 1) were used to amplify approximately 750 bp of *COI* as follows: 94 °C for 4 min, followed by 30 cycles of 94 °C (50 s), 59 °C (50 s), and 72 °C (90 s), followed by 72 °C for 7 min. Approximately 1500 bp of the 16 s rRNA gene (~ 1500 bp) was amplified in a similar fashion but with a 54 °C annealing temperature. PCR products run on a 1% agarose gel were extracted using a Nucleospin Gel and PCR Clean-up kit (Machery Nagel, Duren, Germany) (Fig. S2). Sanger sequencing of gel-extracted amplicons was carried out by Genetic Analysis Services (University of Otago, New Zealand). Sequence reads from amplified COI and 16 s rRNA genes from CAtmus1PFR gDNA and gDNA extracted from the whole fish were aligned using BioEdit. Where errors became frequent, sequences were truncated. Chromatograms were scanned for miscalls and corrected where deemed appropriate. Using the forward and reverse amplification primers as previously, a consensus sequence of 622 bp resulted for *COI*. For 16 s rRNA, a sequencing primer coupled with the previously used forward primer was used to generate a 694 bp consensus sequence.

**Table 1.** Primer sequences used for DNA barcoding

Primer name	Target	Sequence	Ref
COI-Fish-F^a^	*COI*	TTC TCA ACT AAC CAY AAA GAY ATY GG	Kochzius *et al.* (2010)
COI-Fish-R	*COI*	TAG ACT TCT GGG TGG CCR AAR AAY CA	Kochzius *et al.* (2010)
16fiF140	*16 s rRNA*	CGY AAG GGA AHG CTG AAA	Kochzius *et al.* (2008)
16fiR1524	*16 s rRNA*	CCG GTC TGA ACT CAG ATC ACG TAG	Kochzius *et al.* (2008)
16fiseq1463*	*16 s rRNA*	TGC ACC ATT AGG ATG TCC TGA TCC AAC	Kochzius *et al.* (2008)

*COI* cytochrome c oxidase subunit I, *16 s rRNA* 16 s ribosomal RNA

^*^Sequencing primer

### Effects of FBS, basal media, and temperature on CAtmus1PFR cell growth

**Figure 2.**
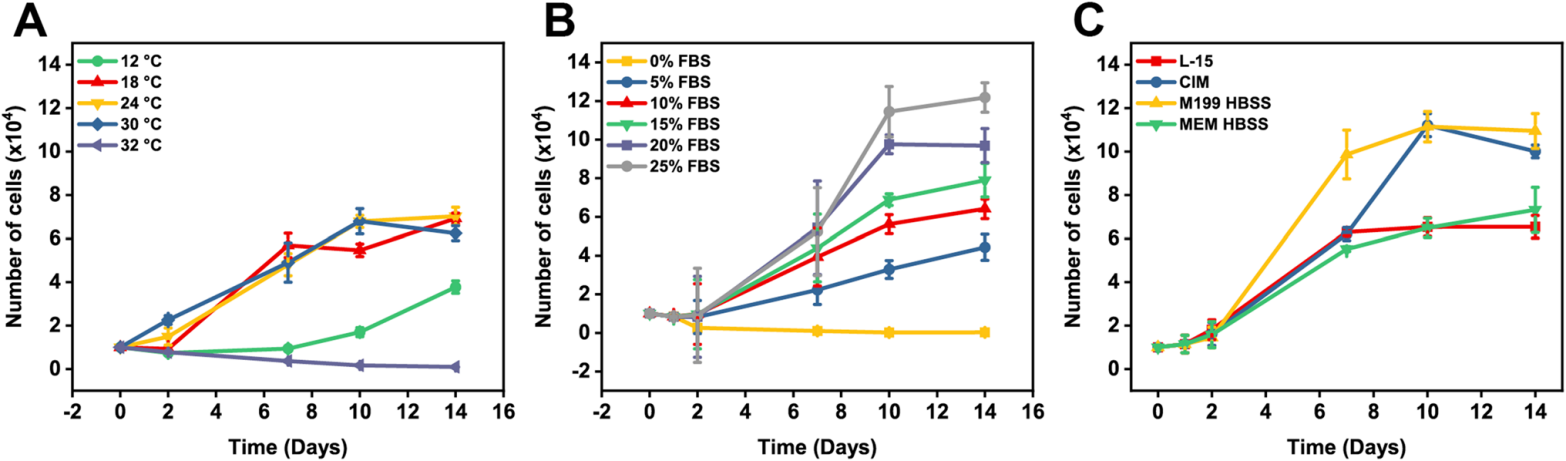
Proliferation of CAtmus1PFR under different parameters. The impact of (*A*) temperature (*B*) FBS and (*C*) basal media on CAtmus1PFR was monitored via direct cell counts over a 14-d period (*n* = 3 for all experiments). *Error bars* = means ± standard deviation.

Cells (1 × 10^4^) were cultured in 24-well plates containing 1 mL of standard culture media (L-15, 1 × P/S, 10% FBS) and incubated at 18 °C for 24 h to allow cell attachment to occur. Following this period, cells were challenged with varying FBS concentrations (0%, 5%, 10%, 15%, 20%, or 25%) or different basal media formulations (L-15, CO_2_ Independent Medium (CIM, Gibco), Medium 199, Hanks’ Balanced Salts (M199-HBSS, Gibco), MEM, Hanks’ Balanced Salts (MEM-HBSS, Gibco) supplemented with 10% FBS and 1 × P/S. At 2, 7, 10, and 14 days post inoculation, cells were detached in 1 × TrypLE Express and counted using a haemocytometer. The impact of temperature on CAtmus1PFR cells was examined by seeding each well of 24-well plates with 1 × 10^4^ cells and incubating immediately at various temperatures (4 °C, 12 °C, 18 °C, 24 °C, 30 °C, 32 °C). Cell number was determined as above. Data shown in Fig. 2 represent data from three biological replicates carried out on different days. Each biological replicate had three technical replicates.

### Impact of growth factors on cell growth

Pilot experiments confirmed the correlation between cell number and measured cell metabolic activity through use of an MTT assay (3-(4,5-dimethylthiazol-2-yl)-2,5-diphenyltetrazolium Bromide, Invitrogen; data not shown), and so, this method was employed to monitor the impact of basic fibroblast growth factor (bFGF, human recombinant, Catalogue No. PHG0266, Thermo Fisher), transforming growth factor beta 1 (TGFβ1, human recombinant, Catalogue No. PHG9204, Thermo Fisher), insulin-like growth factor-1 (IGF-I, human recombinant, Catalogue No. PHG0071, Thermo Fisher), and insulin-like growth factor-2 (IGF-II, human recombinant, Catalogue No. PHG0084, Thermo Fisher) on cell proliferation.

Twenty-four-well plates were seeded at 1 × 10^4^ cells mL^−1^ in standard culture media (L-15, 10% FBS, 1 × P/S) supplemented with 1 or 100 ng mL^−1^ of a single growth factor. All plates were incubated in the dark at 18 °C in ambient air. At 1, 4, 7, and 10 days, an MTT assay was carried out as described previously. Briefly, wells were washed twice with 1 mL phosphate-buffered saline (PBS, Thermo Fisher), followed by addition of 0.4 mL serum-free media supplemented with 0.1 mL MTT (5 mg mL^−1^). Cells were incubated for 70 min at room temperature, after which 0.5 mL DMSO was added to each well. Cells were incubated with agitation for 10 min followed by gentle trituration. A 0.9 mL sample from each well was transferred to a cuvette where the absorbance was measured at 600 nm (Evolution 220 UV–Visible Spectrophotometer, Thermo Fisher). The spectrophotometer was blanked using serum-free L-15, MTT, and DMSO.

### RNA extraction and qPCR

Cells were seeded at 1 × 10^4^ cells mL^−1^ in media (L-15, 10% FBS, 1 × P/S) supplemented with 1 or 100 ng mL^−1^ growth factors (bFGF, IGF-I, IGF-II, or TGFβ1). After 4 days in culture, cells were washed twice in 1 mL dPBS and RNA was extracted and combined from four wells for each condition using a GENEzol TriRNA Pure Kit (Geneaid, New Taipei City, Taiwan) as per the manufacturer’s recommendations. An on-column DNase treatment was carried out to remove genomic DNA. Complementary DNA (cDNA) was synthesised from 500 ng of RNA using Superscript VILO (Invitrogen) in a 20 µL reaction volume. qPCR primers were designed based on the gilthead seabream (*Sparus aurata*) which is closely related to *C. auratus.* Database accession numbers of each target and reference genes are identified in Table 2. All primers were designed to span an exon-exon junction and should, therefore, not detect genomic DNA, sequences of which are shown in Table 2. Primer efficiencies were calculated to be between 91 and 98% (Table 2). Real-time PCR was performed using a Quant Studio 6 Flex instrument (Thermo Fisher). Reactions consisted of 0.5 µL forward and reverse primers (10 µM), 8 µL TB Green® (Takara Bio, Shiga, Japan), 6 µL UP H_2_O, and 1 µL cDNA for a total reaction volume of 16 µL.

**Table 2.** Database accession numbers and primer sequences of each target and reference genes

Gene name	Accession number	Primer name	Primer sequence	Primer length	PCR product size	Predicted Tm	Primer efficiency
*actA2*	XM_030400792.1	SA.ActA2.F1	TCGGTGTCGTAGTGTCGTCG	20	189	61.89	95.8%
		SA.ActA2.F1	ACCCACCATCACTCCCTGGT	20		62.71	
*dcn*	XM_030440315.1	SA.Dcn.F6	AAGAAGTAGAGCGCGTTGCC	20	106	61.01	104.5%
		SA.Dcn.R6	GTCGAACACGCACGCTTAC	19		59.87	
*col1A*	XM_030407011.1	Sa.Col1A.F1	TCTCCCCTCAGATGTCCGGT	20	176	61.88	98.2%
		Sa.Col1A.R1	ACCCATGGCACCAGAAGGTC	20		62.14	
*elfA*	XM_030411990.1	Sa.Elf1.F1	TACCCTCCCCTTGGTCGTTTC	21	173	61.45	91.4%
		Sa.Elf1.R1	CGGCACACTTCTTGTTGCTGG	21		62.59	
*vim*	XM_030404168.1	SA.VIM.F2	CAGGCGGTTACCAAGACACC	20	182	60.95	104%
		SA.VIM.R2	CAGTGGGGTGGTGATTCTGCTC	22		63.15	

Elongation factor 1a (*elfa*) was used as reference gene and the threshold cycle (C_T_) values were between 13 and 14. C_T_ values for *col1A*, *dcn*, *sma*, and *vim* were ~ 16, ~ 23, ~ 16, and ~ 23 in the control conditions respectively. The relative fold change in gene expression was calculated using the delta delta C_T_ method (2^−ΔΔCT^) as described by Livak and Schmittgen (2001).

### F-actin labelling

Cells were seeded at a density of 1 × 10^4^ cells/well in a 24-well plate in the presence or absence of 100 ng mL^−1^ bFGF or TGFβ. After 4 days in culture under standard conditions, wells were washed in dPBS and cells fixed in 4% paraformaldehyde for 30 min at room temperature. Cells were permeabilised in 0.4% Triton-X-100 in dPBS for 20 min followed by treatment with Rhodamine phalloidin (Invitrogen) in 0.1% BSA in dPBS for 1 h. Cells were washed and imaged using fluorescence microscopy (CKX53, Olympus ).

### Migration assay

Cells (1.2 × 10^3^ mL^−1^) suspended in L-15 growth medium were seeded in 0.07 mL volume in each side of a two-well migration chamber (IBIDI, Bavaria, Germany). Growth factors (bFGF or TGFβ) were added to a final concentration of 1 or 100 ng mL^−1^. Chambers were incubated in ambient air and temperature, and the media refreshed on day 4. On day 7, the migration chamber was removed, leaving a 0.5-cm cell-free gap between cell populations. Debris was removed by washing in 1 mL dPBS. Fresh growth factor containing growth media was added, and the plates were imaged immediately using an inverted microscope. Subsequent images of the cell-free gap were recorded at 6 and 24 h post removal from the migration chamber. The gap in a 1500 × 450 pixel (equivalent to ~ 3.5 mm ×  ~ 1 mm) section from each condition was measured using ImageJ software. Each migration assay, of which there were two biological replicates, had triplicate technical replicates.

### Animal handling

All research carried out in this study was reviewed and approved by the Animal Ethics Committee of Nelson Marlborough Institute of Technology in New Zealand (Application number AEC2019-PFR-01).

## Results

### CAtmus1PFR is a continuous cell line derived from *C. auratus* tail skeletal muscle

Collagenase treatment of a muscle segment derived from a juvenile *C. auratus* (Fig. 1*A*) resulted in a preparation with a significant amount of debris. This was not consistent with microbial contamination, but rather appeared to be fragments of degraded muscle fibres (Fig. 1*B*, *C*). By day 9, small colonies of replicating cells with fibroblastic or spindle morphologies were observed (Fig. 1*B*). Many of these early cell types died off and were replaced with other proliferative cells with varying morphologies (Fig. 1*C*, *D*). After 1 month in culture, as the primary culture neared confluency, a heterogenous population of cells was evident with a number of different cell morphologies (Fig. 1*E*, *F*). Following continuous passage, a single cell type emerged which had long spindle-like processes when culturing (Fig. 1*G*). However, on reaching confluency, a subpopulation of cells with a larger, more rounded morphology developed. Cells were successfully cryopreserved and resuscitated in L-15 with 10% FBS and yielded approximately 80–90% cell viability. No morphological changes were observed following resuscitation. At the time of publication, the cell line has been passaged 100 times over a 3-yr period. The line is assumed continuous through spontaneous immortalisation, as is common for finfish cell lines, and has been designated CAtmus1PFR (*Chrysophrys auratus*, tail muscle, Plant and Food Research). The absence of *Mycoplasma* species was confirmed through PCR (Fig. S1). The species origin was verified as *Chrysophrys auratus* by 100% homology between 16s rRNA and COI gene sequence from CAtmus1PFR genomic DNA to gDNA extracted from a *C. auratus* fin clip.

### Temperature, serum concentration, and basal media formulation impact proliferation of CAtmus1PFR

The thermal tolerance of CAtmus1PFR to temperatures ranging from 4 to 32 °C was investigated (Fig. 2*A*). At hypothermic conditions (4 °C), the cells completely detached from the culture vessel after 2 d in culture. At 12 °C, the cells proliferated slowly, and, by day 14, the final numbers were approximately half that of normothermic conditions (18 °C). At 32 °C (hyperthermic), the cells were unable to replicate, but remained attached to the tissue culture vessel. Remaining cells were enlarged in size compared with cells cultured at normothermic conditions (18 °C). Temperatures ranging from 18 to 30 °C provided conditions for comparable growth of CAtmus1PFR cells with no variation in cell morphology (Fig. 2*A*).

In the absence of serum, the number of viable cells decreased over time; by day 14, a small number of cells remained with an elongated morphology (Fig. 2*B* and S3). Decreasing FBS concentration from the standard 10 to 5% resulted in a lower proliferative rate. Conversely, increasing the serum concentration resulted in a dose-dependent increase in cell number over a 14-d period (Fig. 2*B*). In media supplemented with 25% FBS, the cell number after 2 wk in culture was more than double that observed with 10% FBS (Fig. 2*B*). There was no impact on cell morphology with FBS concentrations between 5 and 25%.

CAtmus1PFR is routinely cultured in L-15 media which allows the cells to be cultured without the need for additional CO_2_. Alternative buffered media were investigated for their impact on proliferation of CAtmus1PFR cells (Fig. 2*C*). After 7 days in culture, proliferation of CAtmus1PFR in M199 was significantly greater than that observed in three other basal media preparations (L-15, CO_2_-independent medium [CIM], and MEM HBSS). By day 10, the maximum cell number had been reached in M199 (~ 1.1 × 10^5^), and this was matched by CIM. Interestingly, cells culturing in L-15 and MEM reached maximal number at 7 d (~ 6 × 10^4^), and did not show further growth even with fresh media replacements. Final cell numbers in MEM and L-15 were significantly lower than in CIM and M199.

### Growth factors affect proliferation and morphology of CAtmus1PFR

**Figure 3.**
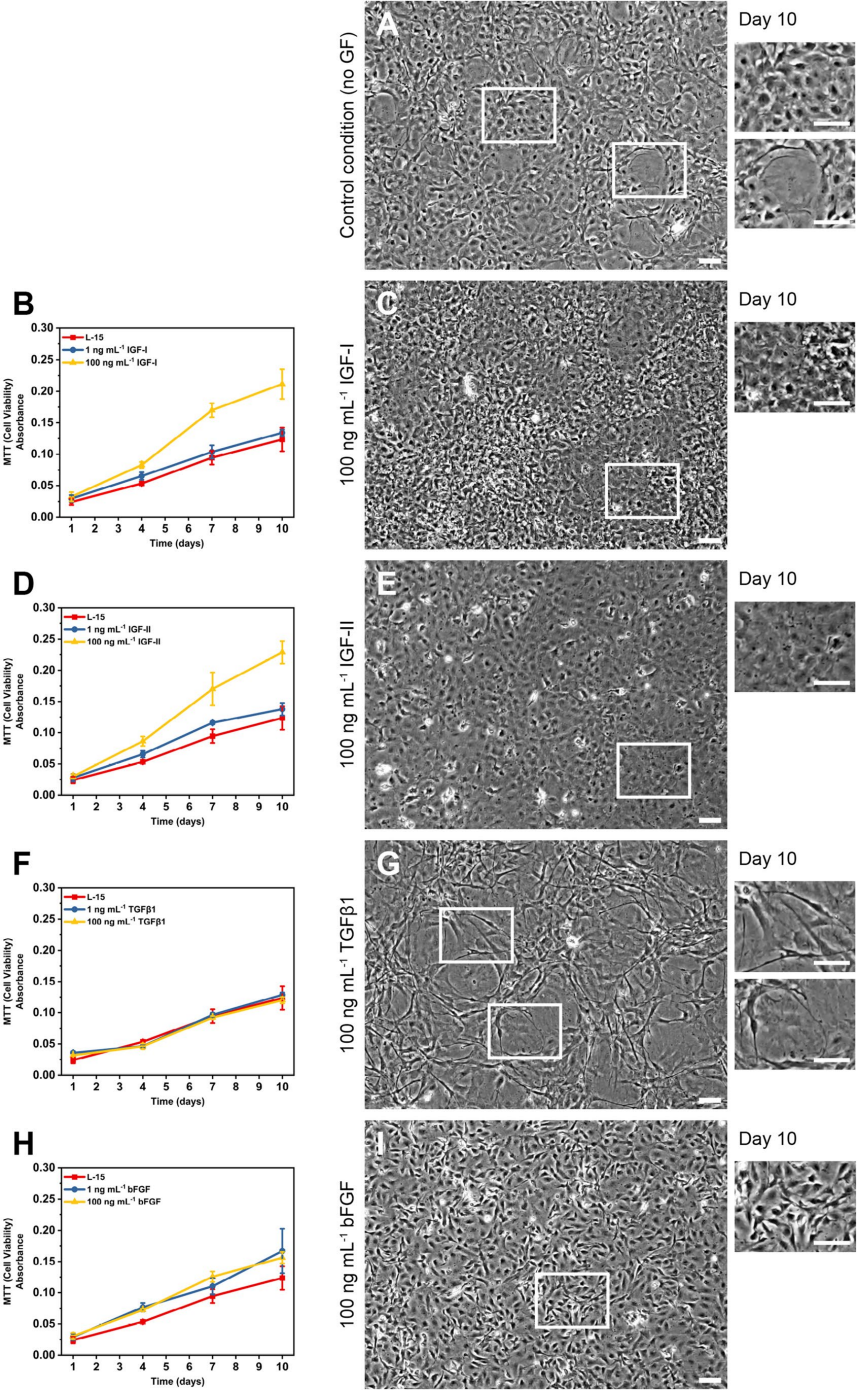
Impact of growth factors on morphology and replication of CAtmus1PFR. (*A*) Micrographs showing pleiomorphic phenotype in control condition (L-15 media supplemented with 10% FBS and 1 × penicillin streptomycin) when confluent. Impact of IGF-I (*B*, *C*), IGF-II (*D*, *E*), TGFβ1 (*E*, *F*), and bFGF (*G*, *H*) on CAtmus1PFR proliferation and morphology at 1 and 100 ng mL^−1^ (*n* = 3). *Error bars* = means ± standard deviation. All* scale bars* are 100 µm.

At 1 ng mL^−1^, insulin-like growth factors I and II (IGF-I, IGF-II) had no impact on cell number as measured by MTT assay; however, at 100 ng mL^−1^, both IGF-I and IGF-II resulted in increased cell numbers (Fig. 3*B*, *D*).

TGFβ1 had no impact on cell numbers, but had a significant impact on the cell morphology (Fig. 3*F*, *G*). The cell size increased and appeared to be similar to the subpopulation of cells observed under standard culture conditions (Fig. 3*A*). bFGF had minimal impact on proliferation of CAtmus1PFR but maintained all the cells in a single morphology (Fig. 3*H*, *I*) similar to those observed in the control condition. The large rounded cells, which formed in the control condition (Fig. 3*A*), did not form in the presence of bFGF (Fig. 3*I*).

### CAtmus1PFR treated with TGFβ have myofibroblast properties

The change in morphology of CAtmus1PFR following treatment with TGFβ1 and bFGF suggested differentiation of the cell. Previous data have demonstrated that TGFβ1 can result in the differentiation of precursor cells to myofibroblasts. These differentiated cells exhibit changes in cell shape, modified cytoskeletal features including the formation of actin bundles, and increased deposition of extracellular matrix components such as collagen. bFGF has been shown to have an opposing effect, causing downregulation of myofibroblastic features and encouraging a more fibroblastic phenotype. We therefore sought to determine if CAtmus1PFR was a myofibroblast progenitor cell, and gave rise to myofibroblasts following TGFβ treatment, and whether bFGF had an opposing effect on this process.

**Figure 4.**
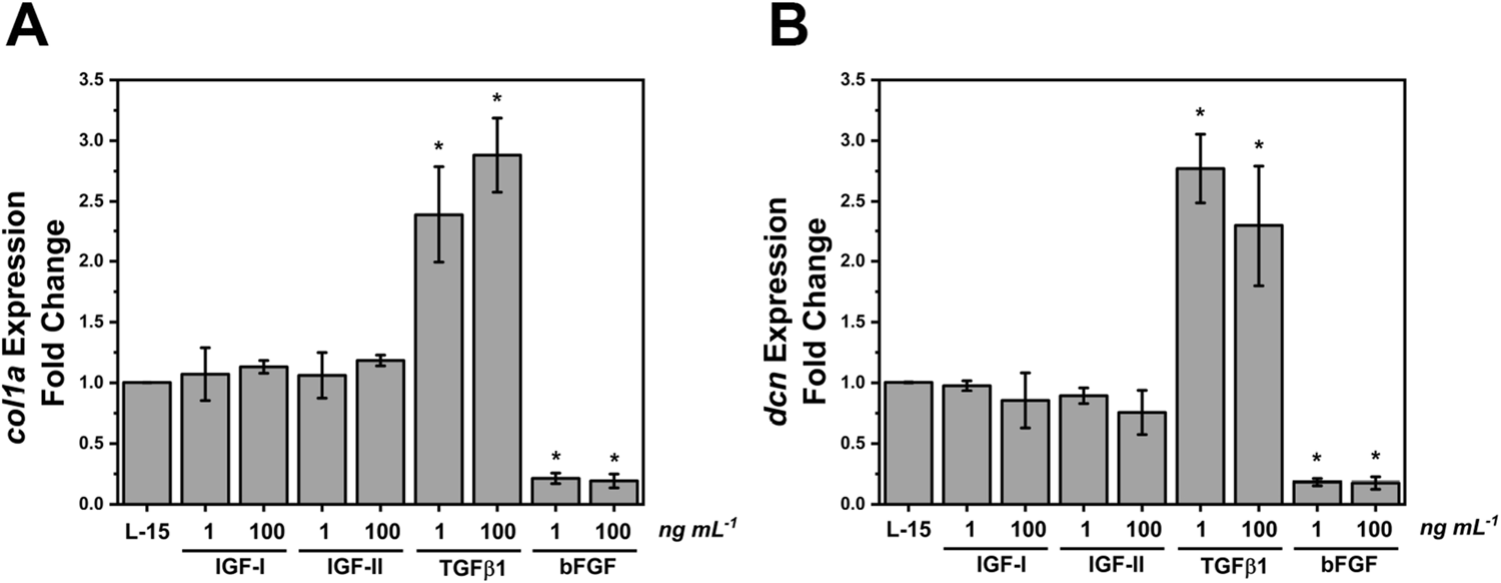
Impact of growth factors on expression of genes associated with extracellular matrix components. Change in expression of (*A*) type 1 collagen 1 (*col1A)* and (*B*) decorin (*dcn)* following 4 d of treatment with IGF-I, IGF-II, TGFβ1, or bFGF at 1 and 100 ng mL^−1^ relative to control condition (L-15, 10% FBS, 1 × P/S) (*n* = 3). *Error bars* = means ± standard deviation. *P < 0.05 by one-way ANOVA followed by Tukey’s least Significant Difference (α = 0.05).

Myofibroblasts are associated in vivo with enhanced deposition of collagen. In TGFβ1-treated cells, a 2.5- to threefold increase in expression of *col1a* mRNA at 1 and 100 ng mL^−1^, respectively, was observed (Fig. 4*A*). Conversely, exposing cells to 1 or 100 ng mL^−1^ bFGF resulted in a significant decrease in expression. The expression of *decorin* mRNA, from a gene encoding a proteoglycan extracellular matrix (ECM) component, was also increased in the presence of TGFβ1 and decreased where the cells were cultured with bFGF (Fig. 4*B*).

**Figure 5.**
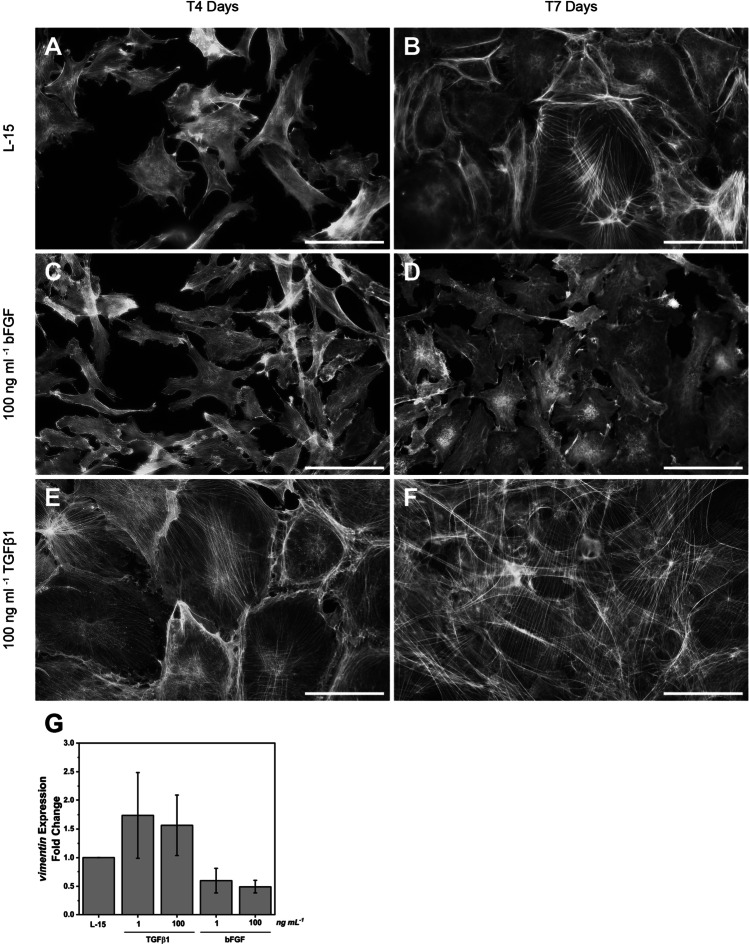
Effects of growth factors on the cytoskeletal structure of CAtmus1PFR cells. Cells 4 and 7 d in culture under standard conditions (*A*, *B*), or in the presence of 100 ng mL^−1^ bFGF (*C*, *D*) or TGFβ1 (*E*, *F*). (*G*) Expression of vimentin after 4 d exposure to TGFβ1 and bFGF at 1 and 100 ng mL^−1^ in comparison to control unexposed cells. *Error bars* = means ± standard deviation. All *scale bars* are 100 µm.

Myofibroblasts have been shown to exhibit actin bundling, which can be visualised with the fluorescent probe phalloidin, which binds to filamentous actin (F-actin). Under standard culture conditions (no growth factors), large cells with actin bundling and smaller cells without bundling were evident after 7 d in culture (Fig. 5*B*). In the presence of bFGF, no actin bundling was observed in any of the cells and the size and morphology remained unchanged (Fig. 5*C*, *D*). In the presence of TGFβ, cells exhibited significant actin bundling and large cell size by 4 d in culture (Fig. 5*E*). By day 7, the cells formed a mesh of cells with significant actin bundling (Fig. 5*F*). An additional cytoskeletal marker, the intermediate filament protein, vimentin, which is known to be expressed by myofibroblasts, was also upregulated following TGFβ1 treatment of CAtmus1PFR cells and downregulated in the presence of bFGF, although the changes were not statistically significant (Fig. 5*G*).

**Figure 6.**
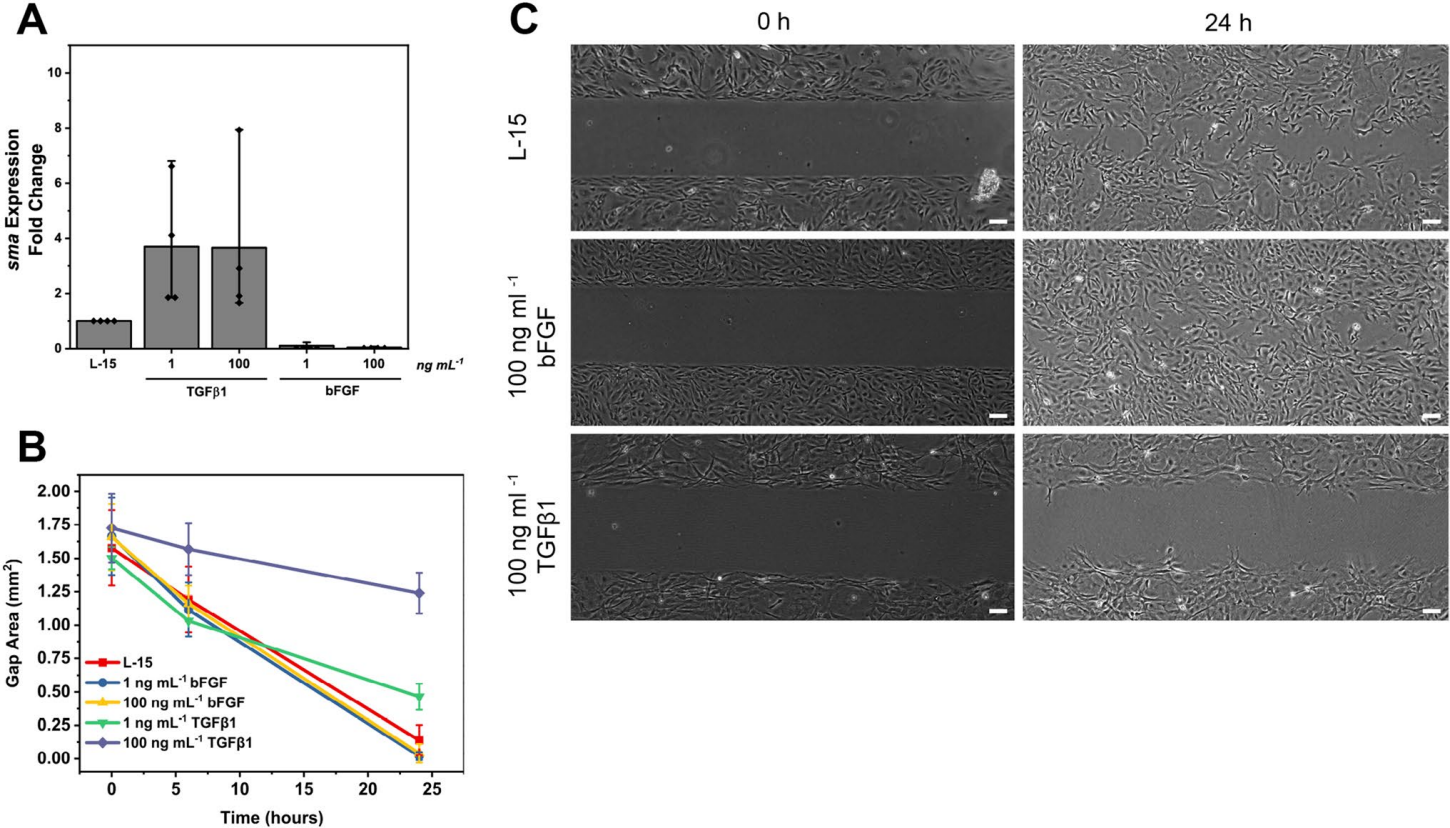
Effects of growth factors on expression of smooth muscle actin (*sma*) and on cell migration. (*A*) Expression of *sma* after 4 d exposure to TGFβ and bFGF at 1 and 100 ng mL^−1^ in comparison to control unexposed cells. Symbols represent fold change from 4 independent biological replicates for each condition. *Bars* represent the mean value for each condition. (*B*) Measurement of gap size at initial ‘wounding’ and 24 h later for cells cultured in standard culture media (L-15, 10% FBS, 1 × P/S), or media supplemented with bFGF or TGFβ at 1 and 100 ng mL^−1^. Values represent means and standard deviations of triplicate biological replicates, each with two or three technical replicates. (*D*) Micrographs demonstrating migration of CAtmus1PFR cultured in control and growth factor-supplemented media over 24 h. *Scale bars* are 100 µm.

Expression of smooth muscle actin (SMA) is generally indicative of myofibroblast differentiation, and, when exposed to TGFβ1, expression of this gene increases in CAtmus1PFR (Fig. 6*A*). A large variation in this increased expression was observed across biological replicates (changes between 2- and eightfold). It is plausible that the status of the seeding culture plays an important role in the expression profile of *sma*, as cultures which have a proportion of cells already differentiated to myofibroblasts may result in an increased expression of sma in the cell population. Conversely, bFGF-treated cells decrease expression of *sma* mRNA (Fig. 6*A*). SMA protein has previously been shown to be involved with reducing the migratory capabilities of cells. To further investigate the migratory capabilities of growth factor–treated cells, a wound-migration assay was carried out. Mobility of cells treated with TGFβ1 was almost completely abolished at 100 ng mL^−1^, and was somewhat retarded at 1 ng mL^−1^ (Fig. 6*C*, *D*). Untreated and cells treated with bFGF migrated into and closed the gap within 24–36 h (Fig. 6*C*, *D*).

## Discussion

Here we describe the growth characteristics and differentiation capacity of a muscle-derived myofibroblastic precursor cell line from the tail peduncle muscle of Australasian snapper. This is the first reported cell line for *C. auratus*, and it joins 26 other cell lines derived from fish skeletal muscle reported in the scientific literature. Of the previously reported lines, the majority are described as being fibroblastic according to their morphology. In many cases, these have been developed as in vitro systems for monitoring and control of infectious disease in aquaculture (Middlebrooks *et al.* 1981; Zhao *et al.* 2003; Zhao and Lu 2006; Lai *et al.* 2008; Wang *et al.* 2020). Despite the emphasis on fibroblasts, skeletal muscle is a complex environment with many cell types, each playing an important role in the development, repair, and homeostasis of muscle tissue (Joe *et al.* 2010; Uezumi *et al.* 2011; Heredia *et al.* 2013; Giordani *et al.* 2019). Additionally, the ECM supporting these cells provides both the physical and biochemical environments in which these cells reside (Csapo *et al.* 2020). Satellite cells, long considered muscle progenitor cells, are abundant in fish (Johnston 2006; Ruparelia *et al.* 2020), and have been shown to undergo dichotomic differentiation into myoblastic and non-myoblastic lineages (Shefer *et al.* 2004). The myoblastic lineage leads to myogenic development, whereas the alternative leads to a mesenchymal pathway giving rise to cells which reside in the connective tissue of muscle. Additionally, mesenchymal stem cells distinct from satellite cells have also been described to occur in skeletal muscle tissues. Work is ongoing to determine the specific stem cell origins that has given rise to CAtmus1PFR.

Fish cell lines have been described as having a proliferation zone with an optimum temperature where cells replicate more rapidly (Bols *et al.* 1992). In the case of CAtmus1PFR, there was a broad optimal temperature where the cells had a similar proliferation capacity (between 18 and 30 °C). This is in comparison to a number of other cell lines where a single temperature is identified as being optimal. A number of rainbow trout cell lines (RTE, RTE-2, RTG-2, RTH-149, and RTT) share an optimal temperature of 20 °C, with decreased proliferation at 5 °C above or below this temperature (Bols *et al.* 1992). The broad temperature tolerance of CAtmus1PFR may be due to the eurythermal nature of snapper (Parsons *et al.* 2014), suggesting cell lines from this species represent the in vivo nature of the fish. It would be of interest to determine if the effect of temperature on the gene expression profile of CAtmus1PFR mirrors that observed in snapper in vivo, where pathways associated with metabolism, cell regulation, and cell signalling were implicated (Wellenreuther *et al.* 2019).

The specific nutritional requirements of skeletal muscle-derived fish cell lines are undefined. Basal media formulations developed for mammalian cells supplemented with mammalian serum as a source of growth factors, lipids, and additional nutrients are routinely used (Bols and Lee 1994). Proliferation of many muscle-derived fish cell lines is positively affected by increasing serum concentrations (Zhao *et al.* 2003; Zhao and Lu 2006; Rougee *et al.* 2007; Wang *et al.* 2020), and this is similar to that observed with CAtmus1PFR. Interestingly, different fish species have different tolerances to basal media formulations (Fernandez *et al.* 1993; Wang *et al.* 2003; Zhao and Lu 2006). Here we have demonstrated CAtmus1PFR is capable of enhanced replication in M199 and CIM media formulations; however, as the impact(s) these media had on the biochemical and physical responses of cells was not determined, we continued to culture the cell line in the medium in which it was established (L-15). It is unclear at this stage whether media preferences are determined by the donor fish species, the donor tissue, or a specific cell type. A comparison of requirements between these variables may provide additional information when it comes to understanding the development and growth of fish muscle for applications in both traditional and cellular aquaculture (Rubio *et al.* 2019).

In fish skeletal muscle, mesenchymal progenitor cells are believed to give rise to myogenic and non-myogenic lineages. The myogenic lineage leads to the formation of skeletal muscle per se, while the non-myogenic lineage leads to myofibroblast generation and is also proposed to form the resident fibroblasts, fibro/adipogenic progenitor cells, pericytes, and stem cells (Paylor *et al.* 2011). The impacts of various growth factors on fish myogenic cells have been reasonably understood, but their effects on the non-myogenic lineage less so. Here we provide evidence that CAtmus1PFR is a non-myogenic mesenchymal progenitor cell that is capable of differentiation to a myofibroblast.

Under normal physiological conditions, many components of the ECM, including type 1 collagen, are produced by muscle-resident fibroblasts. In situations when muscle tissue becomes damaged, myofibroblasts are recruited. These specialised cells increase deposition of collagen, which aids in the repair of muscle tissue, but where the action of these cells is not resolved, a thickening or scarring of the tissue can occur (fibrosis). Decorin, another component of the ECM, prevents the formation of fibrosis by binding to TGFβ, yet there is evidence that myofibroblasts express full-length biologically active decorin, which essentially contradicts the role myofibroblasts play in the formation of fibrosis in vivo (Honda and Munakata 2004). The exact reason for this is not clear but it has been proposed that decorin may play different roles as fibrosis progresses (Klingberg *et al.* 2013). It would be interesting to monitor decorin expression over time in CAtmus1PFR and determine if expression of decorin changes.

TGFβ is a known stimulant of myofibroblast differentiation and fibrosis (reviewed in Frangogiannis 2020). The differentiation process increases expression of type 1 collagen (*col1a*), and induces the expression of alpha-smooth muscle actin (*sma*) expression in myofibroblasts (Desmoulière *et al.* 1993), which may participate in the formation of stress fibres, cell contractility, and cell spreading characterising the myofibroblasts phenotype. TGFβ1 also contributes to the upregulation of vimentin expression while blocking myogenesis (Wu *et al.* 2007) and reducing migration. CAtmus1PFR responded similarly to TGFβ1, and these cells are thus prime for studying in vitro fibrosis events.

In vivo, increased deposition of collagen by cardiac myofibroblasts has been shown to play a role in the remodelling of the ECM of the heart in rainbow trout in response to cold acclimation (Johnston and Gillis 2018). This reactive fibrosis leads to a stiffening of the heart, which provides support for the increasing muscle mass of the fish. This has implications in aquaculture, where fish strains are specifically bred for their ability to increase muscle mass rapidly (Olesen *et al.* 2003). Indeed, perivertebral fibrosis, presumably due to the action of myofibroblasts, has been reported in farmed Chinook salmon which has led to spinal deformities, thus reducing the value of this commercially important species. The exact cause of these deformities is as yet unknown, but a number of factors have been proposed, including temperature, toxicities, trauma, and vitamin deficiencies (Munday *et al.* 2016). Indeed, the mycotoxin, aflatoxin B1, has been shown to cause liver fibrosis in trout (Arana *et al.* 2014), so it would be of interest to determine if this, and other toxins, in addition to compounding effects such as temperature stress, affected fibrosis in an in vitro model. Having a model of fibrosis is a useful tool, not only for understanding possible causes of fibrosis, but also for the development of prevention and rescue strategies, assuming the process is dynamic and, therefore, reversible. While myofibroblast precursor cells have previously been isolated and cultured from heart tissue of rainbow trout (Johnston and Gillis 2018), this is the first time a precursor cell capable of in vitro differentiation has been isolated from fish muscle. Beyond applications in traditional aquaculture, availability of this non-myogenic lineage from snapper tail muscle could also be relevant to the blossoming cellular aquaculture industry where the main focus has been in culturing myogenic cells, but as fish meat texture and structure are heavily influenced by the myoseptum and ECM, myofibroblasts and their precursors may also prove essential for this technology.

## Supplementary Information

Below is the link to the electronic supplementary material.

Supplementary file1 (JPG 327 KB)

Supplementary file2 (JPG 439 KB)

Supplementary file3 (JPG 765 KB)
