# Supplementary figures and images for: A continuous myofibroblast precursor cell line from the tail muscle of Australasian snapper (Chrysophrys auratus) that responds to transforming growth factor beta and fibroblast growth factor
Source: In Vitro Cell Dev Biol Anim. 2022 Nov 15;58(10):922–35. doi: 10.1007/s11626-022-00734-2 (PMC9780137; doi:10.1007/s11626-022-00734-2)

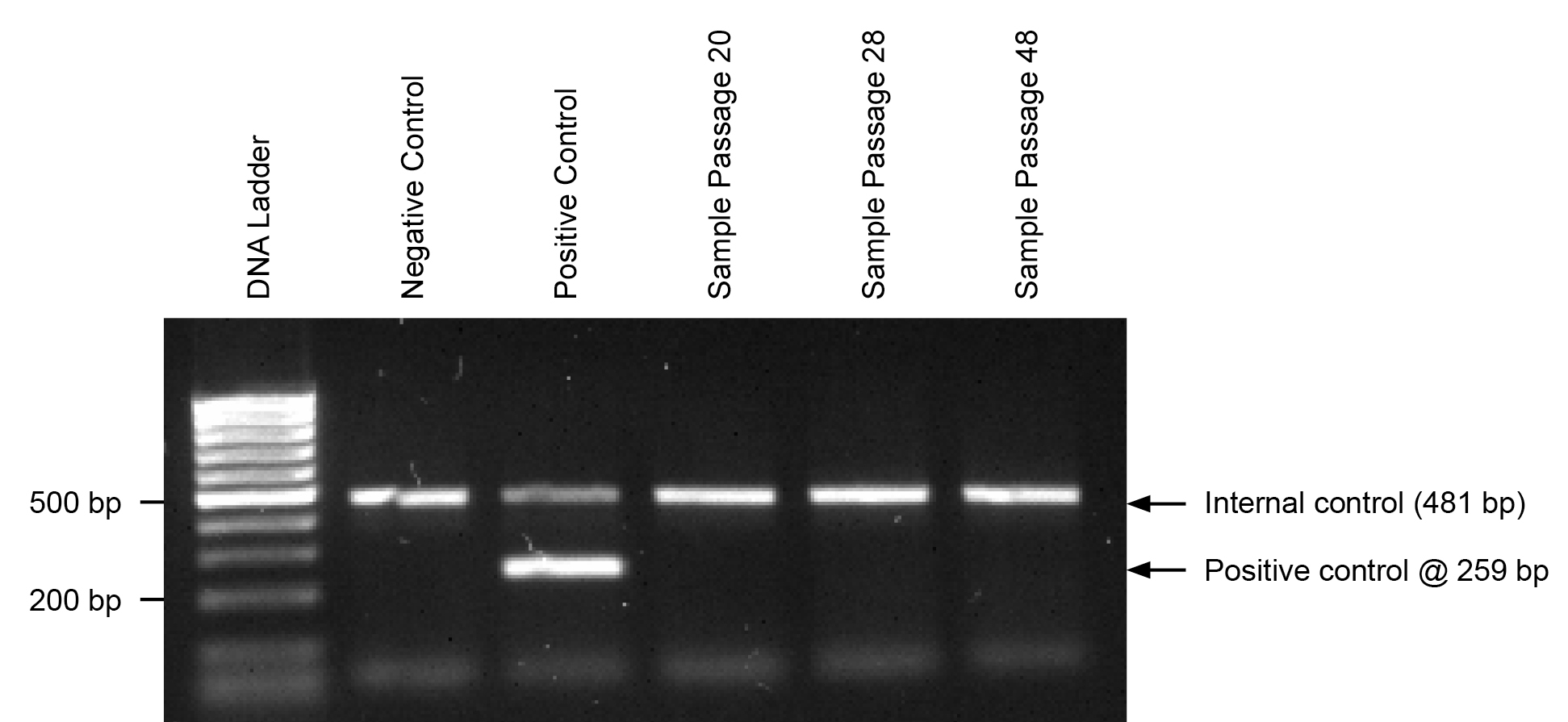

Supplement: Supplementary file 1 — Supplementary file1 (JPG 327 KB) [file 11626_2022_734_MOESM1_ESM.jpg]

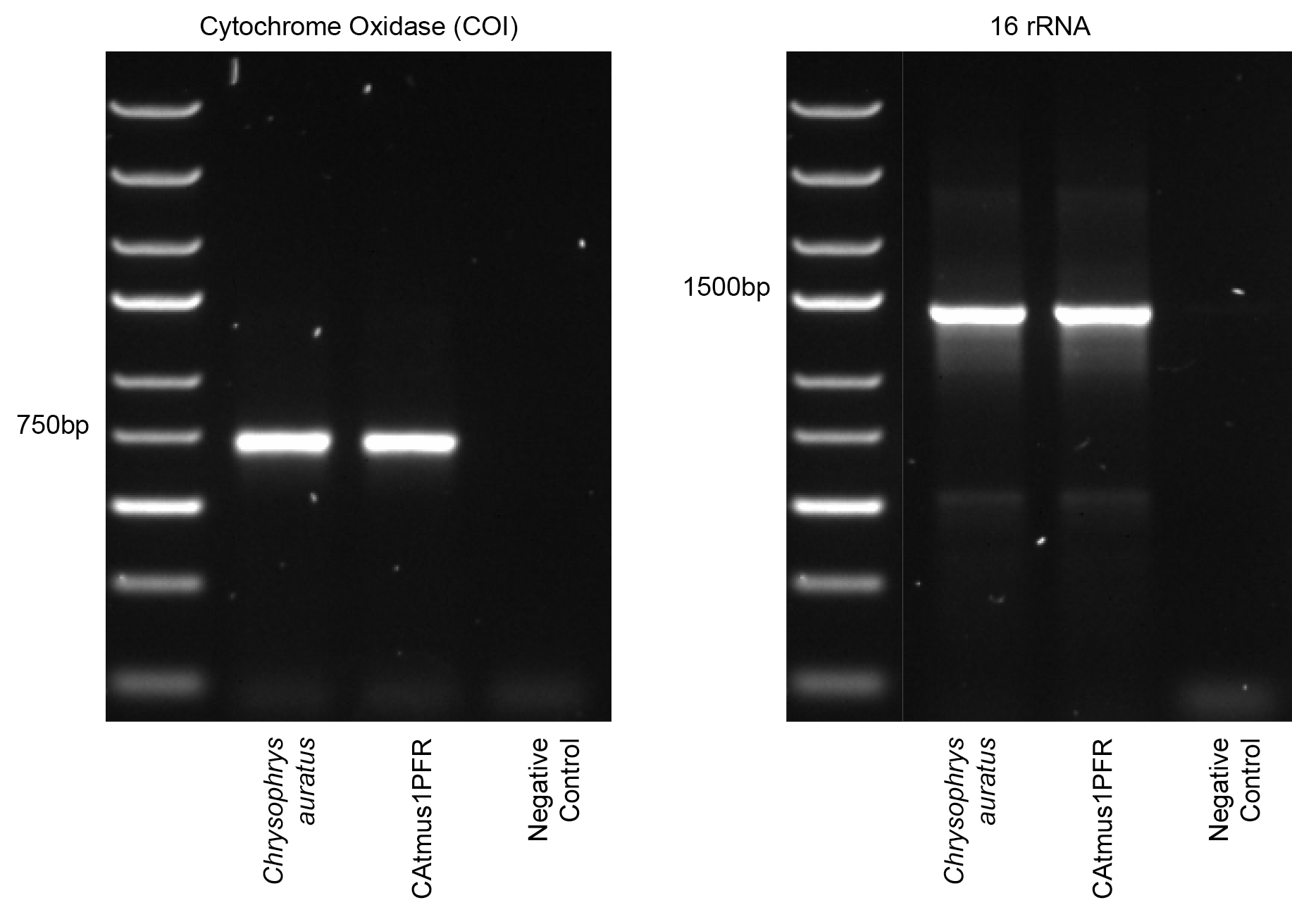

Supplement: Supplementary file 2 — Supplementary file2 (JPG 439 KB) [file 11626_2022_734_MOESM2_ESM.jpg]

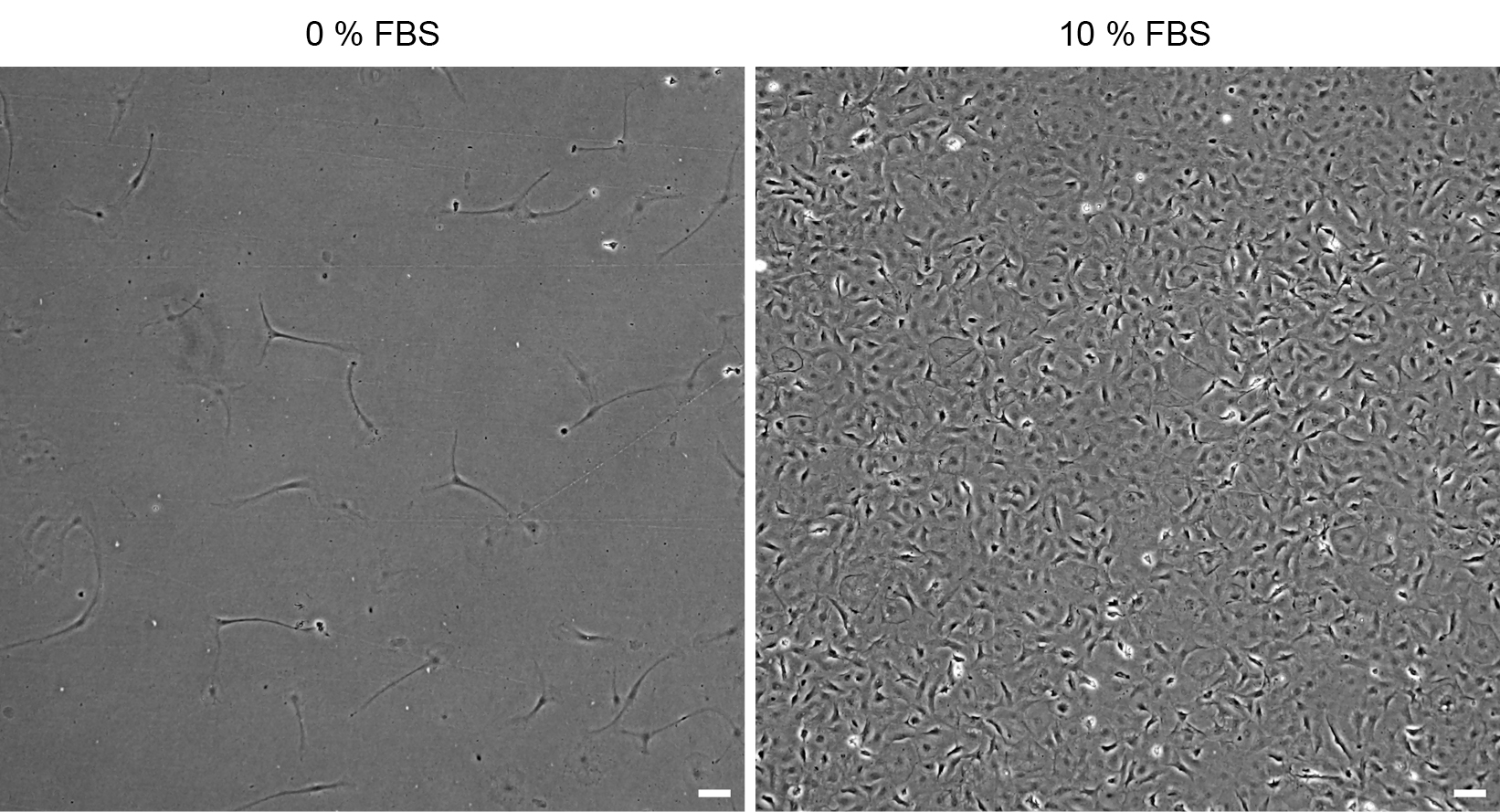

Supplement: Supplementary file 3 — Supplementary file3 (JPG 765 KB) [file 11626_2022_734_MOESM3_ESM.jpg]
